# Antigenic Relatedness of Norovirus GII.4 Variants Determined by Human Challenge Sera

**DOI:** 10.1371/journal.pone.0124945

**Published:** 2015-04-27

**Authors:** Ying-Chun Dai, Xu-Fu Zhang, Ming Xia, Ming Tan, Christina Quigley, Wen Lei, Hao Fang, Weiming Zhong, Bonita Lee, Xiaoli Pang, Jun Nie, Xi Jiang

**Affiliations:** 1 Department of Epidemiology, School of Public Health and Tropical Medicine, Southern Medical University, Guangzhou, Guangdong, China; 2 School of Traditional Chinese Medicine, Southern Medical University, Guangzhou, Guangdong, China; 3 Divisions of Infectious Diseases, Cincinnati Children’s Hospital Medical Center, Cincinnati, Ohio, United States of America; 4 Department of Pediatrics, University of Cincinnati College of Medicine, Cincinnati, Ohio, United States of America; 5 Provincial Laboratory for Public Health (ProvLab), Edmonton, Alberta, Canada; 6 Department of Laboratory Medicine and Pathology, University of Alberta, Edmonton,Alberta, Canada; University of Parma, ITALY

## Abstract

The GII.4 noroviruses (NoVs) are a single genotype that is responsible for over 50% of NoV gastroenteritis epidemics worldwide. However, GII.4 NoVs have been found to undergo antigenic drifts, likely selected by host herd immunity, which raises an issue for vaccine strategies against NoVs. We previously characterized GII.4 NoV antigenic variations and found significant levels of antigenic relatedness among different GII.4 variants. Further characterization of the genetic and antigenic relatedness of recent GII.4 variants (2008b and 2010 cluster) was performed in this study. The amino acid sequences of the receptor binding interfaces were highly conserved among all GII.4 variants from the past two decades. Using serum samples from patients enrolled in a GII.4 virus challenge study, significant cross-reactivity between major GII.4 variants from 1998 to 2012 was observed using enzyme-linked immunosorbent assays and HBGA receptor blocking assays. The overall abilities of GII.4 NoVs to bind to the A/B/H HBGAs were maintained while their binding affinities to individual ABH antigens varied. These results highlight the importance of human HBGAs in NoV evolution and how conserved antigenic types impact vaccine development against GII.4 variants.

## Introduction

Noroviruses (NoVs) are the leading cause of non-bacterial acute gastroenteritis in both developed and developing countries and are responsible for over 50% of NoV acute gastroenteritis outbreaks in the European countries and the United States [1–3]. Although NoV infection is generally self-limited with mild to moderate symptoms, severe morbidity and occasional mortality have been observed in immune compromised patients and the elderly [4,5]. Norovirus infection also results in over 1 million hospitalizations; with ~900,000 clinic visits and ~200,000 deaths of children under 5 years of age in developing countries annually [6]. Norovirus is highly contagious, with a very low infective dose of about 1000 viral particles [7,8], and usually causes large outbreaks within closed communal or institutional settings, such as hospitals, nursing homes, schools, childcare centers, restaurants, cruise ships, and the military [2,9,10]. No vaccines or antiviral therapies have been approved for prevention or treatment of NoV infections, and it has been difficult to study human NoVs due to the lack of an efficient cell culture system or small animal model [11,12]. Due to the high disease burden of NoVs, an effective vaccine is in high demand for high-risk populations, including the young, the immunocompromised, and the elderly.

Noroviruses are single-stranded positive-sense RNA viruses. The genome consists of three open reading frames (ORFs), of which ORF2 encodes the major capsid protein (VP1) [13]. Expression of the ORF2 major recombinant capsid protein *in vitro* successfully produces empty viral-like particles (VLPs), which are morphologically and antigenically similar to native viral particles, and have been used as surrogates for NoV in many studies [14–16]. VP1 can be divided into two major domains: the shell (S) and the protruding (P) domains [13]. The P domain is the most hyper variable region of the genome and is also responsible for carbohydrate ligand binding. Our previous studies showed that expression of the P domain alone resulted in P particle formation, which displayed similar HBGA binding patterns as VLPs and elicited similar innate, humoral, and cellular immune responses as VLPs in mice [17]. P particles are useful research tools for studying NoV and its interactions with carbohydrate ligands [18–20].

Noroviruses are divided into six genogroups (GI to GVI) [13,21]. GI and GII are responsible for most human infections and can be further divided into at least 9 and 22 different genotypes, respectively [22]. A single genotype, GII.4, is recognized as the most prevalent genotype worldwide [23]. Molecular surveillance also showed that GII.4 NoVs undergo constant genetic drifts with new GII.4 variants emerging every one to three years, which coincides with new epidemics of GII.4 acute gastroenteritis worldwide [24–28]. Over the past two decades, at least 9 GII.4 variants have been reported, including GII.4-1996 (Grimsby), GII.4-2002 (Farmington Hills), GII.4-2004 (Hunter), GII.4-2006a (Laurens), GII.4-2006b (Minerva), GII.4-2008a (OC07138/Jap), GII.4-2008b (Appledorn), GII.4-2010 (New Orleans) and GII.4-2012 (Sydney) [10,24–26,29–31]. These variants were estimated to be responsible for 55%-85% of NoV-associated outbreaks in various countries [3].

Genetic variation of GII.4 NoVs is believed to be a result of the low fidelity of the viral polymerase, which is driven by pressure from host herd immunity and leads to GII.4 immune-escape mutants, similar to the epochal evolution of influenza virus [32,33]. Monoclonal antibodies have been useful for recognizing antigenic variations; in addition, monoclonal antibodies that recognize shared antigenic types for all GII.4 variants have been identified [32,34]. Our previous study showed that while the P domain of the NoV capsid protein VP1 was highly variable, the major HBGA binding interfaces of GII.4 variants were highly conserved [35]. Using paired acute and convalescent serum samples from GII.4-infected patients, we also detected significant levels of cross-reactive antibodies among major GII.4 variants [35]. To better understand the antigenic relatedness of GII.4 variants, which should significantly benefit NoV vaccine development, further characterization of the antigenic relatedness of recently emerged GII.4 variants (2008b and 2010 clusters) was performed in this study. Paired pre- and post-challenge serum samples from GII.4-infected patients involved in a human challenge study [36] were used to characterize these recent GII.4 variants. Although continual genetic drifting was observed for the recent variants, as with previous GII.4 variants, their major antigenic and HBGA binding profiles remained un-changed, supporting our previous findings [35]. The potential mechanism of genetic and antigenic conservation and variation of GII.4 variants selected by the host HBGAs and herd immunity is also discussed.

## Materials and Methods

### Ethics Statement

Human stool sample collection was approved by the Research Ethics Board of the University of Alberta, Canada (Pro00037093). Human serum sample collection was approved by the institutional review boards of the Cincinnati Children’s Hospital Medical Center (protocol # IACUC2013-0128) and the US Army Medical Research and Materiel Command’s Human Research Protection Office. Subject consent specific to this study was not necessary, as these were previously collected samples from patients who had consented that their samples could be used in future studies. Immunization of mice was approved by the Institutional Animal Care and Use Committee (IACUC) of the Cincinnati Children’s Hospital Research Foundation (Animal Welfare Assurance No. IACUC2013-0128).

### Stool samples, sequencing and phylogenetic analysis

The antigenicity and HBGA binding profiles of GII.4 NoVs identified in stool samples from patients in the province of Alberta, Canada from 2000 to 2012 were further characterized. Viral RNA was extracted from 10% stool suspensions with the QIAamp Viral RNA Mini Kit (Qiagen, Valencia, CA). The P domain coding cDNAs were amplified using the Qiagen one-step RT-PCR kit (Valencia, CA), then sequenced after the amplified cDNAs were cloned into pGEM T-vectors. Multiple sequence alignments of the GII.4 variant P domain sequences were performed using Clustal X (version 1.83) [37]. Phylogenetic trees of selected GII.4 variants were constructed using the neighbor-joining method in MEGA (version 4.1) [38].

### Preparation of P particles

The P proteins of different GII.4 strains were made as described previously [19]. A cysteine-containing peptide was linked to the N (CNGRC-P) or C (P-CDCRGDCFC) terminus of the P domains to enhance P-particle formation. The cDNAs encoding the capsid P domain without the hinge were cloned into the expression vector pGEX-4T-1 (Amersham Biosciences, Piscataway, NJ) between Bam HI and Not I sites. After sequence confirmation, the P proteins were expressed in *E*. *coli* following previously described procedures [35,39,40]. Briefly, the BL21 cultures were induced by IPTG (isopropyl-β-D-thiogalactopyranoside) (0.4 mM) at room temperature (22°C) overnight. The recombinant P domain-GST fusion proteins were purified using Glutathione Sepharose 4 Fast Flow resin (GE Healthcare life Sciences, NJ, USA) according to the manufacturer’s instructions. GST was removed from the P proteins by thrombin (GE Healthcare life Sciences, NJ, USA) cleavage on beads at room temperature overnight. The P-particle formation was confirmed by gel filtration, using a Superdex 200 (GE Healthcare Life-Sciences, Piscataway, NJ) size exclusion column, during which the P particles formed a peak at ~830 kDa [18].

### Immunization of mice with GII.4 P particles

The animals were housed in a temperature-controlled environment with 12 h light/dark cycles and received food and water under the control of CCHMC Veterinary Services (Cincinnati Children’s Hospital Medical Center). Five different GII.4variant P particles (VA387, 2007Y2008bC, 2010Y2008bC, 2011Y2010C4, and 2012Y2010C2) were used to immunize mice. The BALB/c mice (2 mice per group) were immunized with P particles plus alum adjuvant (Invitrogen Life Technologies Carlsbad, CA, USA) (3:1, volume: volume) subcutaneously four times at 2-week intervals. Sixty μg of P particles were used for the first immunization and then 30 μg for the remaining three boosts. The mice were anesthetized by Isoflurane inhalation, then blood samples were collected using cardiac puncture and the mice were sacrificed.

### Cross-reactive IgG detection in human sera

Paired serum samples collected from patients at day 0 (pre-challenge) and day 30 after GII.4 NoV challenge [36] were used to study the antigenic relatedness among different GII.4 variants by enzyme-linked immunosorbent assays (ELISA). Briefly, 96-well plates (Dynex Immulon; Dynatech, Franklin, MA) were coated with 0.5 μg/ml P particles diluted in PBS at 4°C overnight, then blocked with 5% nonfat dry milk at 37°C for 1 hr. Paired patient serum samples (day 0 and day 30) from the GII.4 NoV human challenge study were added to the plates after 2-fold serial dilution in 2% nonfat dry milk. After a1-hour incubation at 37°C, horseradish peroxidase (HRP)-conjugated rabbit anti-human IgG in 2% nonfat dry milk was used to detect the presence of NoV antibodies. The signals were developed using a TMB substrate kit (Kierkegaard and Perry Laboratory, Gaithersburg, MD).

### HBGA binding assay

P particle binding to HBGAs was measured by saliva- and synthetic oligosaccharide-based binding assays. The saliva samples were selected from a previously described panel of saliva with well-defined A, B, O, and Lewis antigens [15,35]. Boiled saliva samples were diluted 1:1000 with 1XPBS and coated onto 96-well microtiter plates at 4°C overnight. After blocking with 5% nonfat dry milk, P particles (20 μg/ml) were added after 3-fold serial dilution and incubated at 37°C for 1 hr. The bound P particles were detected using guinea pig anti-NoV (1:3330) sera, followed by HRP-conjugated goat anti-guinea pig IgG (ICN, Aurora, OH). The signals were developed using a TMB substrate kit.

For the oligosaccharide-based binding assays, microtiter plates were coated with recombinant P particles (2 μg/ml) at 4°C overnight. After blocking with 5% Blotto nonfat milk, oligosaccharide-polyacrylamide (PAA)-biotin conjugates (2 μg/ml) were added and incubated at 4°C overnight. Oligosaccharides used in this study included H type 1, H type 2, H type 3, type A and type B disaccharides, type A and type B trisaccharides, Le^a^, Le^x^, Le^b^, Le^y^, and sialic-Le^a^ and sialic-Le^x^ tetrasaccharides (GlycoTech Corporation, Rockville, MD). Bound oligosaccharides were detected by HRP-conjugated streptavidin (Jackson Immuno Research Laboratories Inc., West Grove, PA).

### HBGA blockade assay by sera from GII.4 NoV human challenge study

A blocking assay to measure the ability of serum antibodies to block NoV P particles from binding to H type 3 synthetic carbohydrates was developed and optimized. NoV P particles (0.5–1.5 μg/ml) were coated onto 96-well micro-titer plates at 4°C overnight. After blocking with 5% nonfat milk, paired serum samples from the GII.4 NoV human challenge study were diluted in 2% nonfatmilk, added to the plates, and incubated at 37°C for 2 hrs. H type 3-PAA-biotin (2μg/ml) was added and the plates were incubated at 4°C overnight. Bound H3 was detected using HRP-conjugated streptavidin (1:5000) (Jackson Immuno Research Laboratories Inc., West Grove, PA). P particle binding to H type 3 in the absence of a serum sample was used as a positive control. Results were accepted if positive control OD values were within the range of 1.0 ± 0.3. The 50% blocking titer (BT_50_), defined as the titer when the OD reading (after subtraction of the blank) was 50% of the positive control OD value, was determined for each sample. A value of 12.5 was assigned to samples with a BT_50_ of <25 [41].

### HBGA blockade assay with hyperimmune antisera from mice

The saliva binding assays described above were modified to measure the abilities of the hyperimmune antisera collected from immunized mice to block P particles from binding to HBGAs. Briefly, the same format as the saliva binding assay was used, except that the mice antisera were pre-incubated with VA387(98Y96C) P particles (250 ng/ml) in a separate 96-well microtiter plate at 37°C for 1 hour before they were transferred into the saliva-coated plates. The blocking titers at which 50% (BT_50_) or 90% (BT_90_) of the binding was blocked were calculated from the optical density at 450 nm (OD_450_) values between wells with and without incubation with the mice antisera, as described previously [35].

The abilities of hyperimmune mice antisera to block VA387 P particles (0.5 μg/ml) from binding to the H type 3 synthetic oligosaccharides were also studied by blocking assay, as described above.

### Statistical analysis

Graphs were made using Microsoft Office Excel 2010. To test the differences among serum samples in the HBGA blocking assays, we used the Kruskal-Wallis test among groups and the Mann-Whitney test with Bonferroni adjustment between two groups. Paired Wilcoxon test was used to compare antibody titers between pre- and post-challenge sera samples against different P particles. All statistical analyses were performed using SPSS software 13.0 (SPSS, Chicago, Illinois). Differences are considered significant when P<0.05.

## Results

### Phylogenetic analysis of GII.4 variant P domain sequences

A phylogenetic analysis of the P domain amino acid sequences from 82 GII.4 strains circulating between 1974 and 2012 was performed. Forty-six sequences were selected from GenBank, 18 sequences were isolated from samples collected in Canada between 2000 and 2012 in this study, and the remaining18 sequences were from our previous study [35]. The minimum (pairwise) amino acid identity among the 82 sequences was 84.9%.

**Fig 1:**
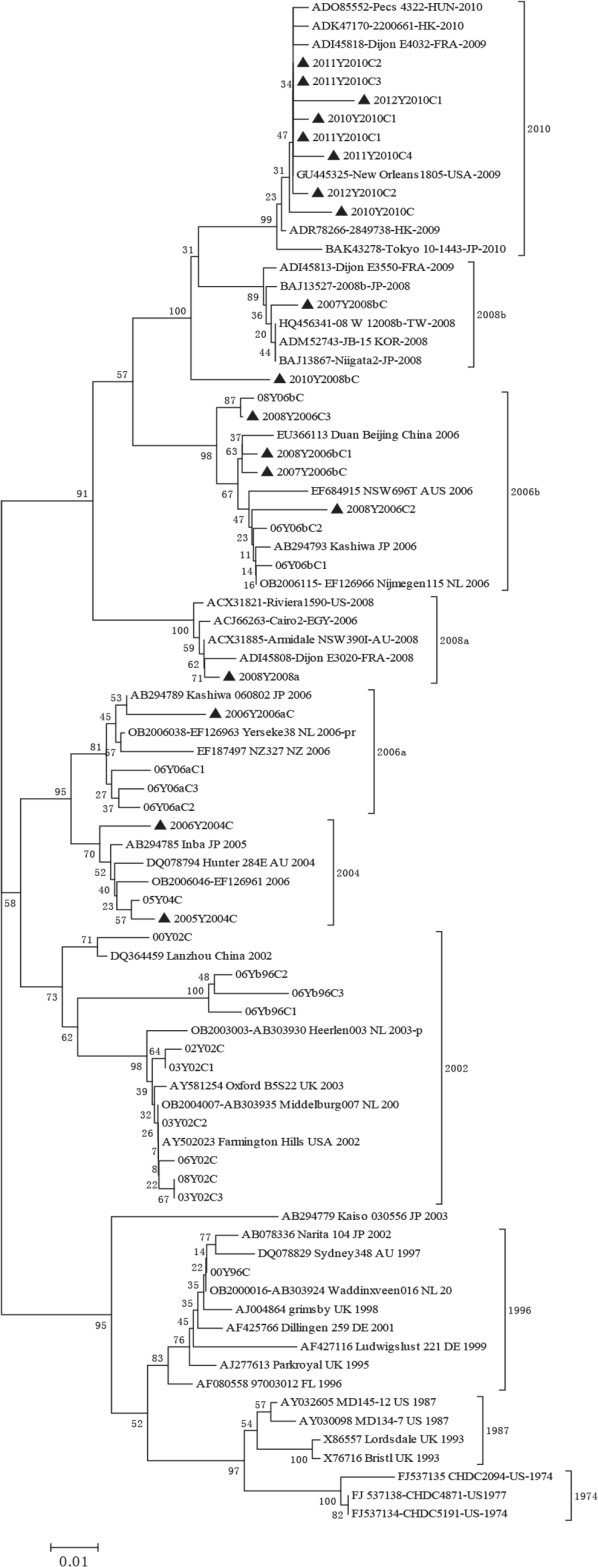
Phylogenetic tree of GII.4 norovirus P domain sequences. Multiple alignments were generated using 82 P domain amino acid sequences with MegAlign Lasergene software and the phylogenetic tree was generated using the neighbor-joining method in MEGA (version 4.1). GII.4 sequences from 1974 to 2012 grouping in different clusters are labeled. Strains isolated in this study are as follows: 2005Y2004C, 2006Y2004C, 2006Y2006aC, 2007Y2006bC, 2008Y2006bC1, 2008Y2006bC2, 2008Y2006bC3, 2008Y2008aC1, 2007Y2008bC, 2010Y2008bC, 2010Y2010C1, 2010Y2010C2, 2011Y2010C1, 2011Y2010C2, 2011Y2010C3, 2011Y2010C4, 2012Y2010C1, and 2012Y2010C2. These strains are highlighted by a solid black triangle.

The 82 GII.4 sequences can be divided into 10 clusters in the phylogenetic tree, which includes the 6 GII.4 clusters (1987-2006b) reported in our previous study [35] and 3 new clusters identified in recent years (2008a, 2008b, and 2010 New Orleans) [25](Fig 1). A nearly GII.4 variant identified in 1974 [42] was also included. The 10 clusters can be further grouped into three major branches: 1) before 1996, 2) 2002 – 2006a, and 3) after 2006b. Each of the three branches includes three to four clusters that are parallel to each other. The three major branches were also parallel to each other without a specific chronological order.

### The HBGA-binding interfaces of GII.4 NoVs are highly conserved

**Fig 2:**
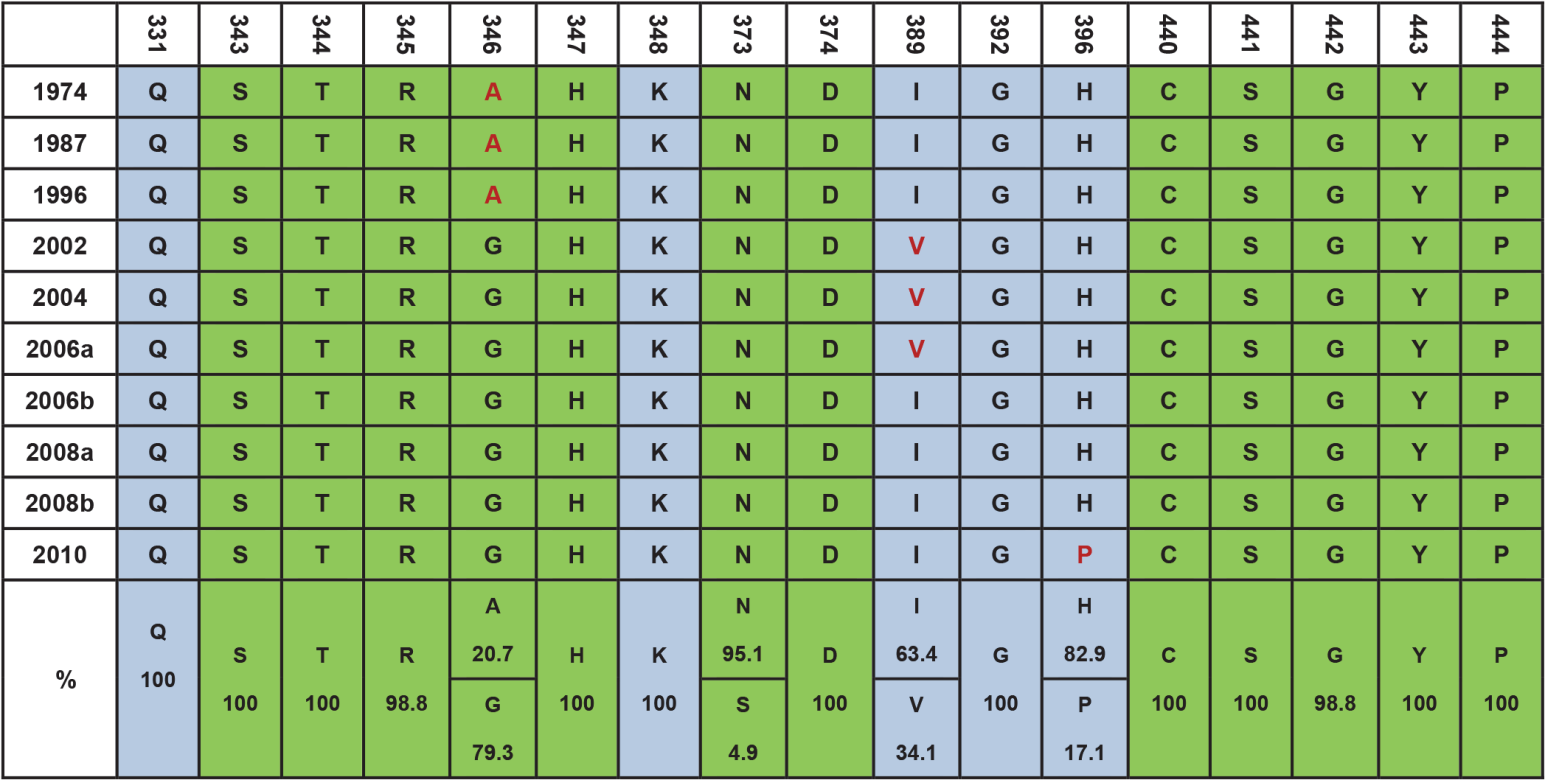
Analysis of HBGA-binding interfaces and the surrounding region for representative GII.4 strains. Ten representative sequences were selected from each major phylogenetic cluster from 1974 to 2012. The P domain sequences were aligned using MegAlign. Amino acids in green indicate residues that are required for HBGA binding. Light blue residues indicate amino acids that affect the binding specificity. Red letters indicate differences from the consensus sequence within the binding interfaces or surrounding regions. The percent similarity between all 82 strains is indicated at the bottom for key amino acids involved in HBGA binding.

Sequence alignment of the 82 GII.4 NoVs revealed highly conserved amino acids scattered in three regions of the P-2 domain of VP1 (region 1: S343, T344, R345, A/G346, and H347; region 2: S373; and region 3: D374, C440, S441, G442, Y443, and P444) (Fig 2). These conserved amino acids form a conformational binding interface interacting with two to three saccharide residues of the ABH antigens according to the crystal structures of selected GII.4 NoVs [43]. Interestingly, amino acids S343, T344, R345, D374, G442, and P444, forming the major binding site that interacts with the H (α-1,2 fucose) epitope of secretor antigens, were almost 100% identical among all 82 GII.4 variants. In addition, most of the amino acids in the surrounding regions were also highly conserved. Only two residues (I389 and H396) revealed minor variations. Residue I389 was found in strains circulating before 2002 then changed to V389 in strains identified between 2002 and 2006, then changed back to I389 after 2006. This reversal between I389 and V389 has been suggested to be associated with changes in binding affinity to the A antigen [35]. Residue H396 was conserved among all GII.4 variants from 1974 to 2010, while cluster 2010, with variants from 2010 to 2012, was the one exception with residue P396.

### The HBGA binding properties of GII.4 P particles are unchanged

**Fig 3:**
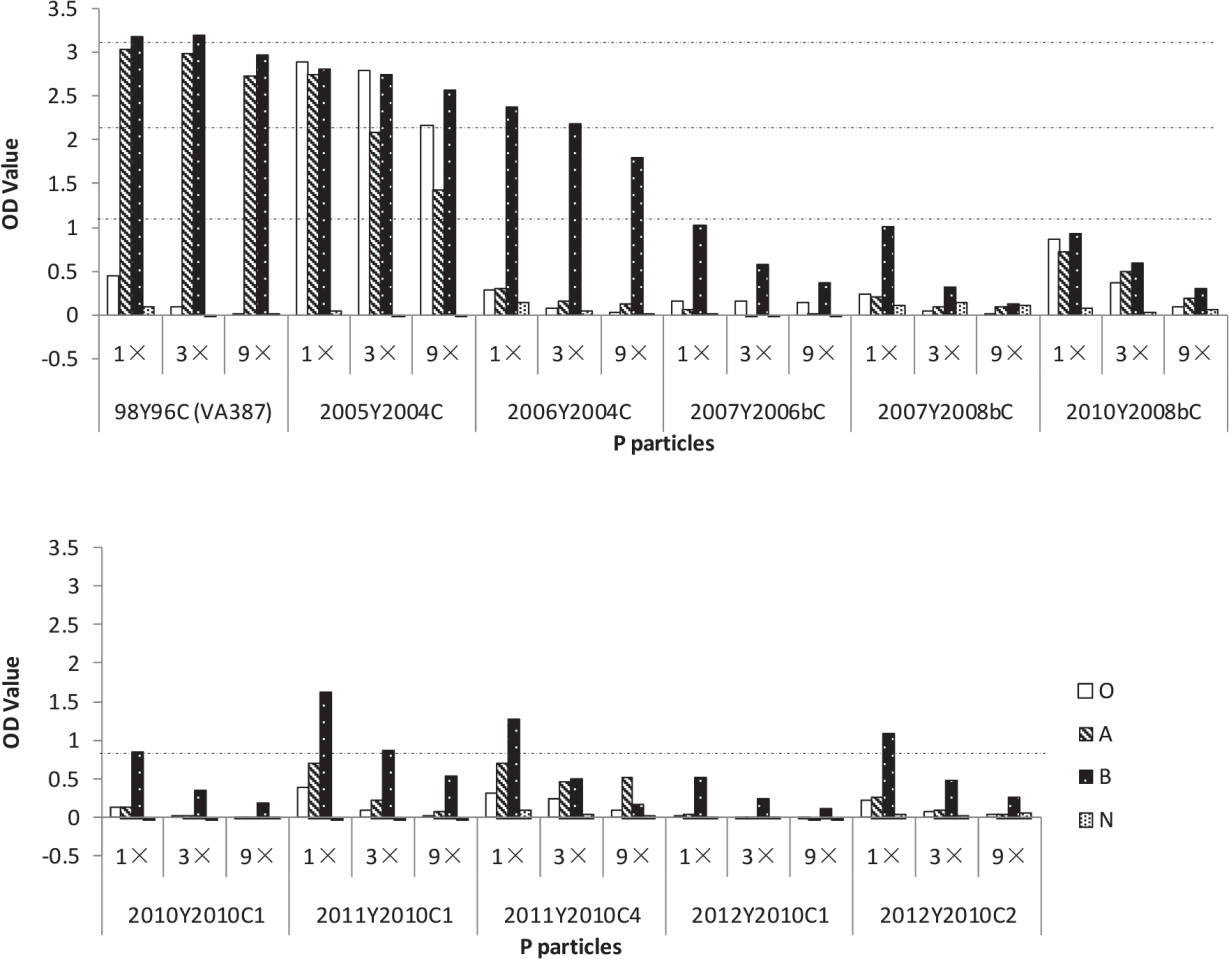
Binding of GII.4 P particles to human saliva samples. Boiled saliva samples were coated onto 96-well plates prior to the addition of P particlesfrom ten GII.4 viruses expressed in this study (2005Y2004C, 2006Y2004C, 2007Y2006bC, 2007Y2008bC, 2010Y2008bC, 2010Y2010C1, 2011Y2010C1, 2011Y2010C4, 2012Y2010C1, and 2012Y2010C2). The P particles were tested in a series of 3-fold dilutions by enzyme-linked immunosorbent assay (ELISA) (optical densities at 450 nm were averaged from at least three independent experiments) and the 98Y96C (VA387) P particle was set as the positive control for binding. “O,” “A,” “B,” and “N” represent the type O (H antigen), A, B, and nonsecretor saliva, respectively.

GII.4 strains representing clusters identified from 2004 to 2012 were further characterized for their HBGA binding variations. These included two strains in the 2004 cluster that were isolated in 2005 and 2006, one strain in the 2006b cluster that was isolated in 2007,two strains in the 2008b cluster that were isolated in 2007 and 2010,and five strains in the 2010 cluster that were isolated between 2010 and 2012 (Fig 3). Four strains 2005Y2004C, 2007Y2008bC, 2010Y2010C1, and 2011Y2010C4 exhibited HBGA binding patterns to saliva of type A, B, and O secretors, while the other six strains bound to B secretors, but none bound to saliva from nonsecretors (Fig 3).

**Fig 4:**
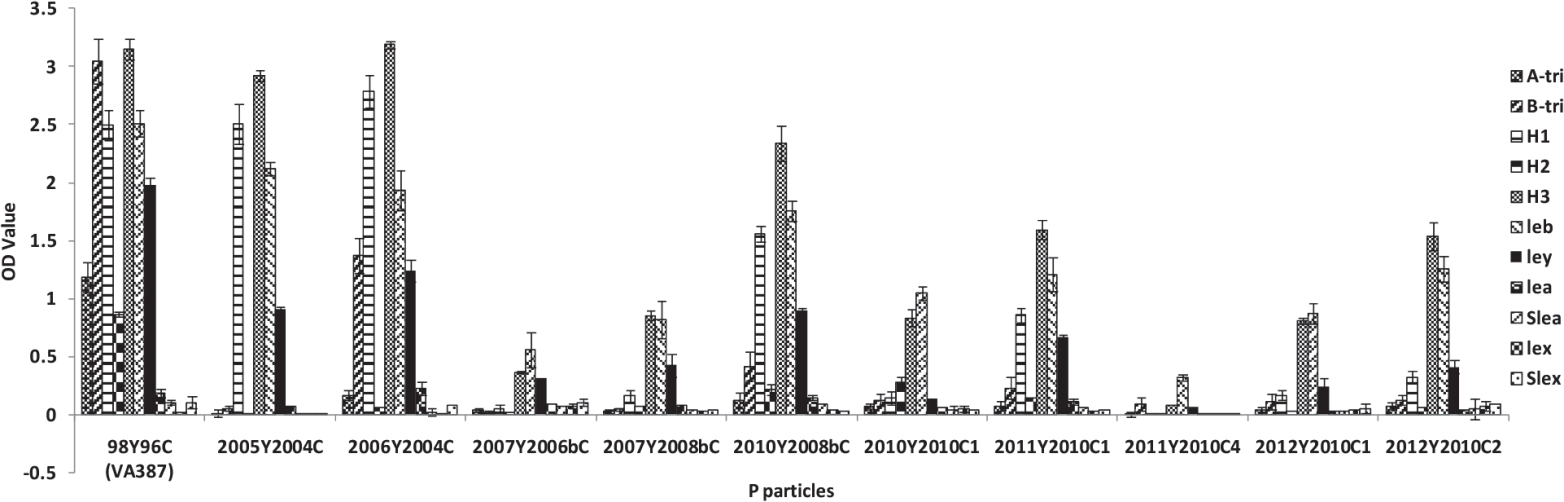
Binding of GII.4 P particles to synthetic oligosaccharides. P particles from 11 strains of GII.4 viruses (2005Y2004C, 2006Y2004C, 2007Y2006bC, 2007Y2008bC, 2010Y2008bC, 2010Y2010C1, 2011Y2010C1, 2011Y2010C4, 2012Y2010C1, and 2012Y2010C2) were coated onto 96-well plates at 2 μg/ml, and then incubated with oligosaccharides (H type 1, H type 2, H type 3, A, B, Le^b^, Le^y^, Le^a^, Le^x^, Sialyl-Le^a^, or Sialyl-Le^x^) overnight. The y-axis indicates captured HBGAs detected by HRP-avidin (optical densities at 450 nm were averaged from at least two independent experiments), and the 98Y96C (VA387) P particle was set as the positive control for binding.

To further define the HBGA binding specificities of the GII.4 viruses, the ten strains were also tested with the oligosaccharide-based binding assay. Nine strains reacted strongly to H-3, Le^b^, and Le^y^ (Fig 4); though 2011Y2010C4 only reacted weakly with Le^b^, it showed good binding to type A, B, O secretors (Fig 3). None reacted with the nonsecretor antigens (Le^a^, Le^x^, sialyl-Le^a^, and sialyl-Le^x^) (Fig 4). Taken together, the ability to recognize the major secretor antigen (α-1, 2 fucose) did not change among most GII.4 variants from 1998 to 2012, although the binding signals to individual carbohydrates varied.

### HBGA-blockade activities using mouse sera

**Fig 5:**
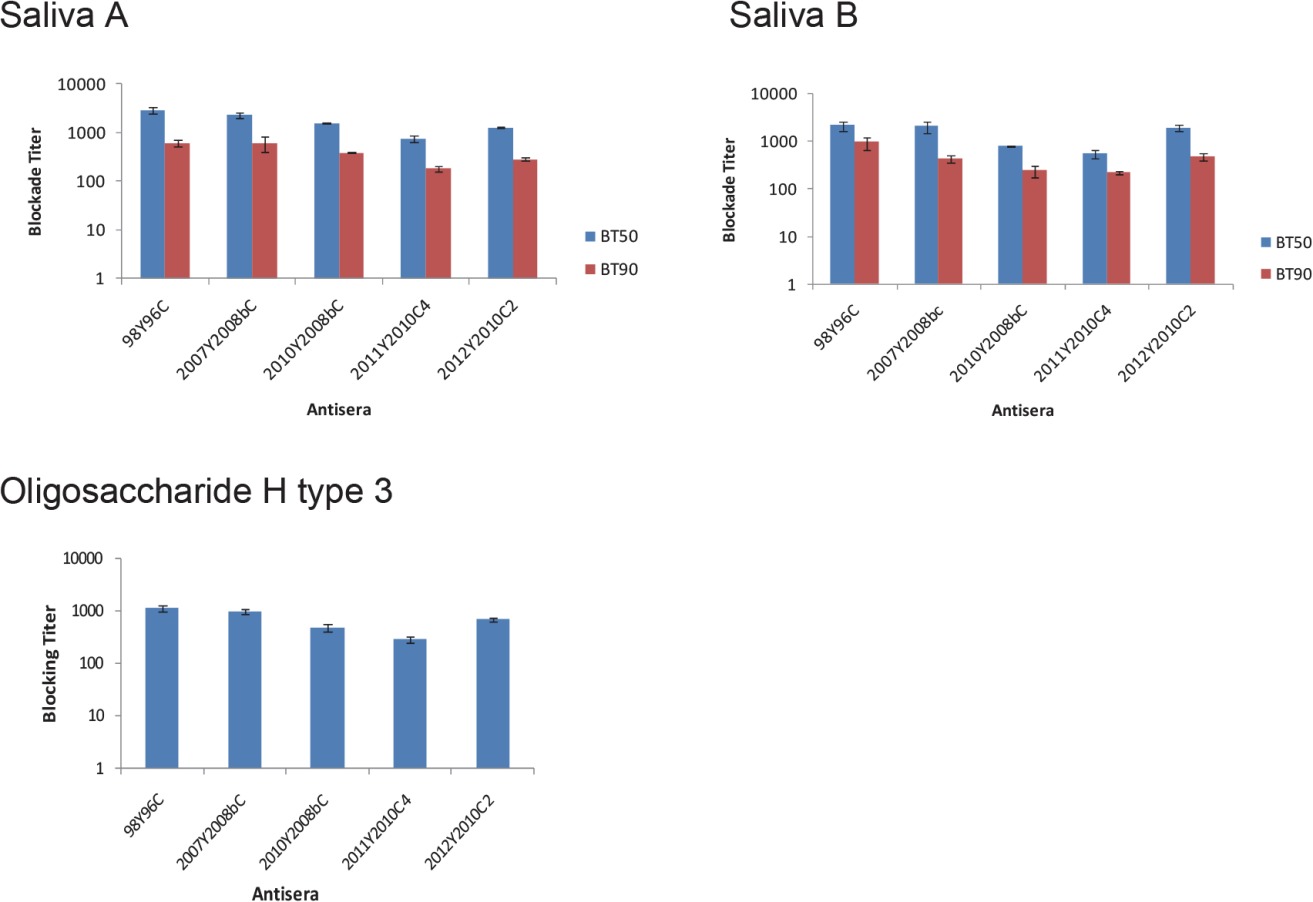
The antibodies from mice immunized with P particles from the 2008b and 2010 clusters blocked GII.4 P particle (VA387, 98Y96C) binding to A-, B-type saliva, and H type 3 oligosaccharide. VA387 P particles were blocked with mouse antisera against each of the five strains (2007Y2008bC, 2010Y2008bC, 2011Y2010C4, 2012Y2010C2, and 98Y96C). The y-axis indicates the serum dilution (blockade titer) as determined from optical density values. The blue bars indicate the antiserum titer at which 50% of the binding was blocked (BT_50_) compared to unblocked controls and red bars indicate the titer at which 90% of the binding was blocked (BT_90_). The results are averaged from at least two independent experiments.

In our previous study, serum samples from mice immunized with P particles of all GII.4 variants from 1996 to 2006 displayed significant cross-blocking activities, inhibiting mouse sera against VA387 P particles from binding to type A (Le^b+^, Le^y+^, and A^+^) and/or type B (Le^b+^, Le^y+^, and B^+^) saliva. In this study, we performed similar blocking experiments, including using mouse sera raised against the recent GII.4 variants 2008b and 2010, and did not observe significant differences in blocking titers among all strains [BT_90_ (χ^2^ = 7.96, P = 0.092) and BT_50_ (χ^2^ = 7.3, P = 0.12)] or between heterotypic vs. homotypic blocking titers against VA387 binding to different secretor antigens (BT_50_: p = 1.000 for 2007Y2008bC vs. VA387; p = 0.484 for 2010Y2008bC vs. VA387, 2011Y2010C4 vs. VA387, and 2012Y2010C2 vs. VA387) (BT_90_: p = 1.0 for 2007Y2008bC vs. VA387; p = 0.484 for 2010Y2008bC vs. VA387, 2011Y2010C4 vs. VA387, and 2012Y2010C2 vs. VA387, Mann-Whitney test with Bonferroni adjustment) (Fig 5).

### Antigenic relatedness among GII.4 variants by ELISA using sera from GII.4-challenged patients

**Fig 6:**
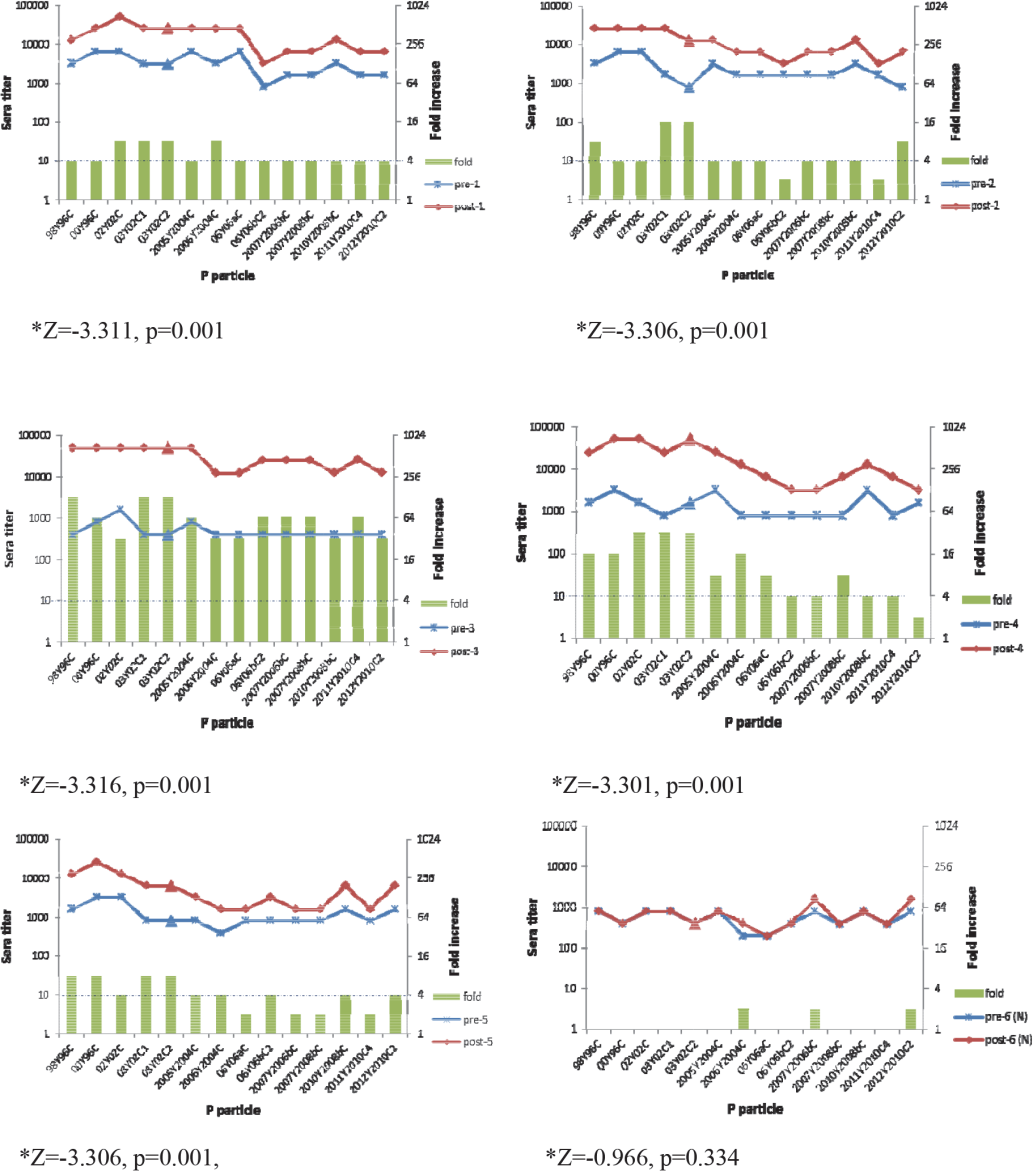
Human sera IgG responses from GII.4 NoV challenge study (03Y02C2, cluster 2002) to GII.4 P particles of different clusters from 1998 to 2012. Pre- (day 0) and post- (day 30) serum samples were collected during a human challenge study. P particles (98Y96C, 00Y96C, 02Y02C, 03Y02C1, 03Y02C2, 2005Y2004C, 2006Y2004C, 06Y06aC1, 06Y06bC2, 2007Y2006bC, 2007Y2008bC, 2010Y2008bC, 2011Y2010C4, and 2012Y2010C2) were coated at 0.5 μg/ml. The fold increases from pre- to post- challenge phase serum in IgG titers (green columns) were determined (right-hand y-axis), and the dashed line indicates fold increases of ≥4. Serum from a non-secretor after GII.4 NoV challenge was set as the control; homology responses to 03Y02C2 are shown with triangle marker. Significant increases (p<0.05) in blocking titer between pre- and post-challenge sera samples were determined for different P particles using the Wilcoxon test.

The antigenic relatedness among different GII.4 variants was further studied using paired pre- (day 0) and post- (day 30) challenge sera from patients involved in a challenge study with a GII.4 NoV(03Y02C2, JQ965810) belonging to the GII.4/2002 cluster [36]. Serum samples from six volunteers (five secretors and one nonsecretor) were tested for antibodies against P particles of 14 GII.4 viruses isolated between 1998 and 2012. Low antibody titers against all14 P particles were detected in the pre-challenge sera (day 0), however, the antibody titers were significantly increased in the convalescent sera (day 30) for all five secretors with seroconversion (≥4-fold increase in titers) against all 14 GII.4 P particles in two subjects (subjects 1 and 3) and 71%-93% of the 14 GII.4 P particles in the other three subjects (Fig 6). Low antibody titers were detected in both the pre- and post- challenge sera of the non-secretor without indication of serocoversion against any of the 14 GII.4 NoVs (Fig 6).

Subject 3 had the strongest responses (128-fold increase) with a low level of pre-challenge antibodies (Fig 6), indicating a primary response, instead of a memory response, against the challenge GII.4 NoV. In addition, strong antibody responses (32 to 128-fold increases) for subject 3 were detected against the other 13 GII.4 NoVs (Fig 6). These results were in accordance with our previous report [35], implying a broad antigenic type exists among GII.4 NoVs circulating from 1998 to 2012.

### Antigenic relatedness among GII.4 variants by HBGA blocking assays using human sera from GII.4 challenge patients

We also tested the post-challenge serum samples for their abilities to block the binding of GII.4 P particles to the H type 3 synthetic oligosaccharide, one of the major secretor HBGAs recognized by GII.4 NoVs (Fig 4). Significant increases in blocking titers were observed between the pre- and post-challenge sera samples against the binding of different P particles (p<0.05) to H3 (Table 1). No significant difference was found among blocking titers of the post-challenge sera against different P particles (χ^2^ = 3.5, p = 0.478, Kruskal-Wallis test). In addition, no significant differences were detected among blocking titers of post-challenge sera when compared to homotypic strain 03Y02C2 (P = 0.756 for 98Y96C vs. 03Y02C2; P = 0.772 for 05Y04C vs. 03Y02C2; P = 0.40 for 06Y06aC vs. 03Y02C2; P = 1.0 for 2010Y2008bC vs. 03Y02C2; Mann-Whitney test). ≥4-fold increases in blocking titers were observed between pre- and post-challenge sera samples in all five paired secretor sera against the five NoV strains. No substantial (>50%) blocking and no increases in blocking titers were observed with the paired sera from the nonsecretor (data were not shown).

**Table 1:** HBGA fifty-percent blocking titer of sera antibody and titer increase fold at pre- and post- phase with GII.4 NoV challenge^#^.

	98Y96C**	03Y02C2**^a^	05Y04C**	06Y06aC**	2010Y2008bC**
Pre-1	50	12.5	12.5	50	12.5
Post-1	200	400	200	200	100
Increase fold^	4	32	16	4	8
Pre-2	25	12.5	12.5	50	12.5
Post-2	200	200	50	200	100
Increase fold^	8	16	4	4	8
Pre-3	12.5	12.5	12.5	25	12.5
Post-3	400	800	800	400	800
Increase fold^	32	64	64	16	64
Pre-4	12.5	12.5	12.5	25	12.5
Post-4	200	400	200	100	200
Increase fold^	16	32	16	4	16
Pre-5	25	12.5	12.5	25	50
Post-5	200	200	100	100	400
Increase fold^	8	16	8	4	8
*Z	-2.032	-2.041	-2.032	-2.041	-2.032
*P	0.042	0.041	0.042	0.041	0.042

^a^ homology virus challenge strain.

Significant increase in blocking titer between pre- and post-challenge sera samples against different P particle (* P<0.05, ** P<0.01, Wilcoxon test).

^#^ No significant difference among blocking titers of post-challenge sera against different P particle (χ^2^ = 3.5, P = 0.478, Kruskal-Wallis test); no significant difference between blocking titers of post-challenge sera compared to homologous 03Y02C2 (P>0.05, Mann-Whitney test with Bonferroni adjustment).

^ A blocking titer fold increase of ≥4 between pre- and post-challenge sera samples were observed in all 5 pair against these five NoV strain.

The pre-challenge serum from subject 3 also had relatively low blocking titers (12.5–25). The post-challenge serum showed blocking titers with 16 to 64-fold increases against the five strains, indicating a strong blocking reaction against different GII.4 strains isolated from 1998 to 2010.

## Discussion

Through characterization of different GII.4 variants that emerged during the past seven years, in this study we further demonstrated the conservation of the HBGA binding interfaces among GII.4 variants. Sequence alignments further extended the conservation of the HBGA binding interfaces to other GII.4 variants that emerged during the past two decades. Using paired acute and convalescent sera from human subjects involved in a volunteer challenge study of a GII.4 NoV variant, we demonstrated significant cross-reactivities among major GII.4 variants circulating from 1998 to 2012 using enzyme-linked immunosorbent assays (ELISAs) and HBGA receptor blocking assays. These results are encouraging for vaccine development aiming to produce a broadly protective vaccine against many GII.4 NoV variants. However, our results also raised a question about how to interpret our data, since the current literature on GII.4 NoV epochal evolution suggests constant emergence of antigenic variants selected through host immunity. Since NoVs are also selected by host HBGAs, further understanding of NoV evolution by both host immunity and HBGAs is necessary.

According to NoV P domain crystal structures [16,43–48], the HBGA binding interfaces can be divided into a highly conserved central binding pocket (CBP) and a less conserved surrounding region (S1 Fig) [49,50]. The CBP interacts with a common carbohydrate residue as the major binding saccharide (MaBS), which is essential for NoV infectivity. The MaBS is shared among strains within each genogroup (genogroups I and II). In fact, the amino acids that form the CBP are almost 100% conserved among all GII.4 variants, indicating strong selection by the host HBGAs during NoV evolution. In other words, no immune escape variants with amino acid changes creating a nonfunctional CBP could survive due to its essential role in NoV infection. Thus, the conserved CBP could function as a major antigenic determinant important for vaccine development with broad protection against different GII.4 variants.

On the other hand, sequence variations have been found on the NoV capsid surfaces, which may be driven by both host immunity and HBGAs [33]. For example, the amino acids surrounding the CBP are more variable among GII.4 variants, which could be driven by the host HBGAs through a different mechanism. These residues interact with additional saccharides as minor binding saccharides (MiBSs) [16,43,50] to facilitate NoV-HBGA interactions. It is known that the CBPs of GII.4 NoVs interact with the H epitope (α-1,2fucose) as the MaBS, while the surrounding region interacts with the A, B, and/or Lewis epitopes as MiBSs. Such coordinated interactions enable GII.4 NoVs to recognize different HBGA types, and therefore facilitate their spread in different human populations. The complicated distributions of human ABH and Lewis types in the world’s populations allow for new GII.4 variants to continually emerge and spread. However, since such variations could be due to positive selection by the HBGAs or negative selection by host immunity, their impacts on broadly protective vaccine development against different GII.4 variants may be less important.

A further concern about GII.4 epochal evolution research is the approach to characterize antigenic variations using monoclonal antibody-based methods. For example, five variable antigenic regions (A-E) on the GII.4 capsid [32,33] have been identified using monoclonal antibodies against GII.4 variants that emerged at different times (S2 Fig). While these data are valuable in understanding antigenic structures and immune escape mutants, direct evidence that such variants were selected via host immunity and their roles in causing major epidemics of GII.4 gastroenteritis remain lacking because of the potential for positive selection of these epitopes by the host HBGAs, as described above. In fact, our data showed strong cross-reactive immune responses among different GII.4 variants using polyclonal antibodies and the paired human sera from volunteer challenge studies; therefore, it is conceivable to anticipate cross-protection. Thus, our polyclonal antibody-based assay described in this study may serve as a better approach for such antigenic assessments for vaccine development against GII.4 variants.

In conclusion, both host immunity and HBGAs play important roles in NoV evolution and are likely to impact different regions/sequences of the viral capsid through interplay between these two host factors. While the monoclonal antibody-based assays provide valuable information, regarding the antigenic structures and dynamic changes of antigenic types of GII.4 variants, this polyclonal antibody-based study provided additional information regarding the conservation of major antigenic types as a result of selection by host HBGAs. In addition, the region surrounding the CBP is also involved in HBGA interactions, which may lead to additional antigenic variations that are not necessarily selected by host immunity. The extensive cross-blocking effects among different GII.4 variants observed in this study emphasize the importance of HBGAs in NoV evolution and vaccine strategy for broad protection against GII.4 variants. Finally, our understanding of GII.4 NoV antigenic variations remains limited; future studies of additional variants from the past, as well as those that emerge in the future are necessary for better vaccine strategies against NoVs.

## Supporting Information

S1 Fig HBGA-binding interfaces and the surrounding region analysis in a crystal model. Cyan, the highly conserved central binding pocket; Orange, conserved surrounding residues; purple, changing residues.
(TIF)

S2 Fig GII.4 P domain antigenic variation. Key sites predicted to modulate receptor binding interactions and the antigenic profiles of the virus were aligned chronologically.
(TIF)

S1 File Ethical Statement and ARRIVE Guidelines Checklist. (PDF)
