# Supplementary figures and images for: Antigenic Relatedness of Norovirus GII.4 Variants Determined by Human Challenge Sera
Source: PLoS One. 2015 Apr 27;10(4):e0124945. doi: 10.1371/journal.pone.0124945 (PMC4411064; doi:10.1371/journal.pone.0124945)

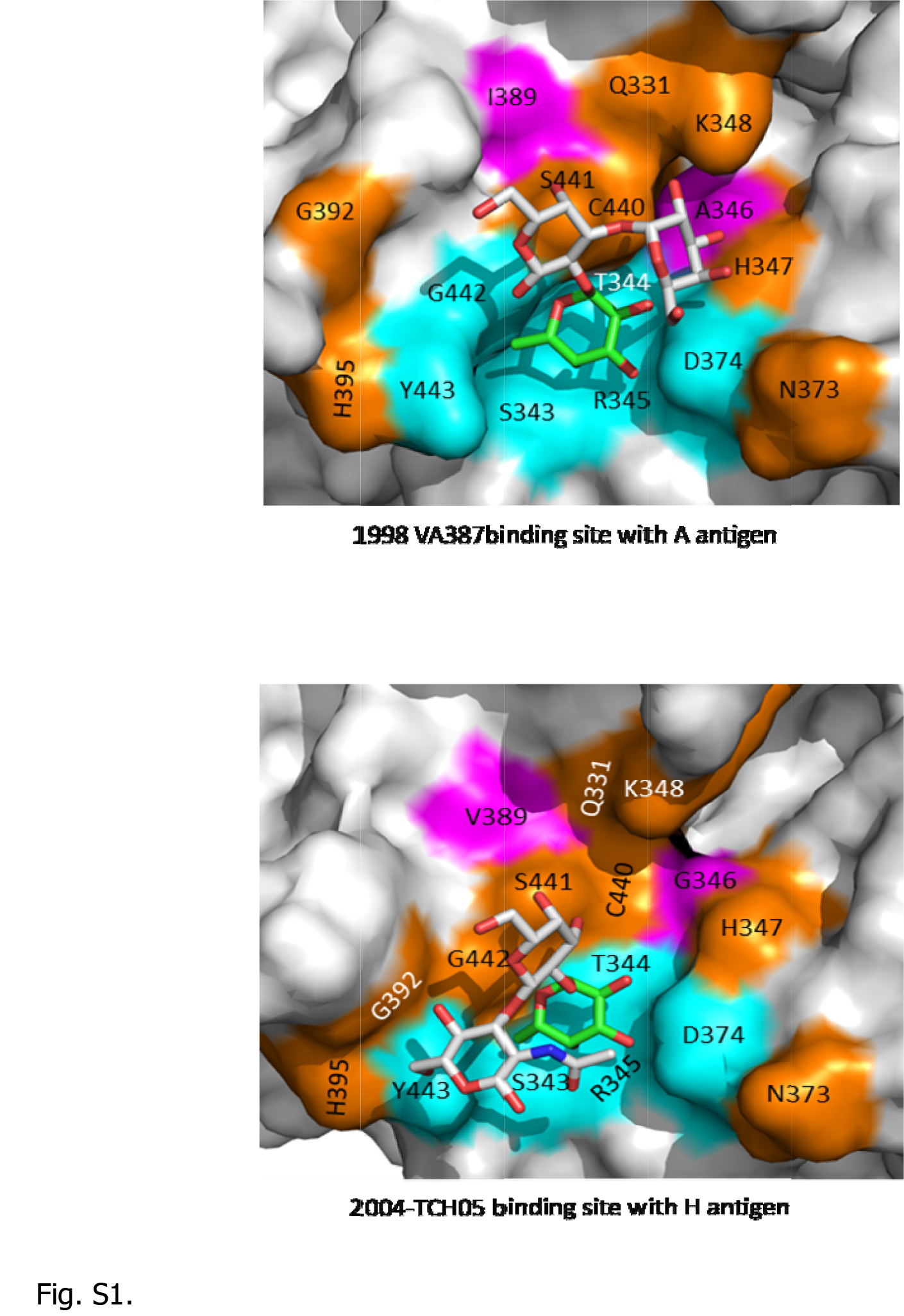

Supplement: S1 Fig — Cyan, the highly conserved central binding pocket; Orange, conserved surrounding residues; purple, changing residues. (TIF) [file pone.0124945.s001.tif]

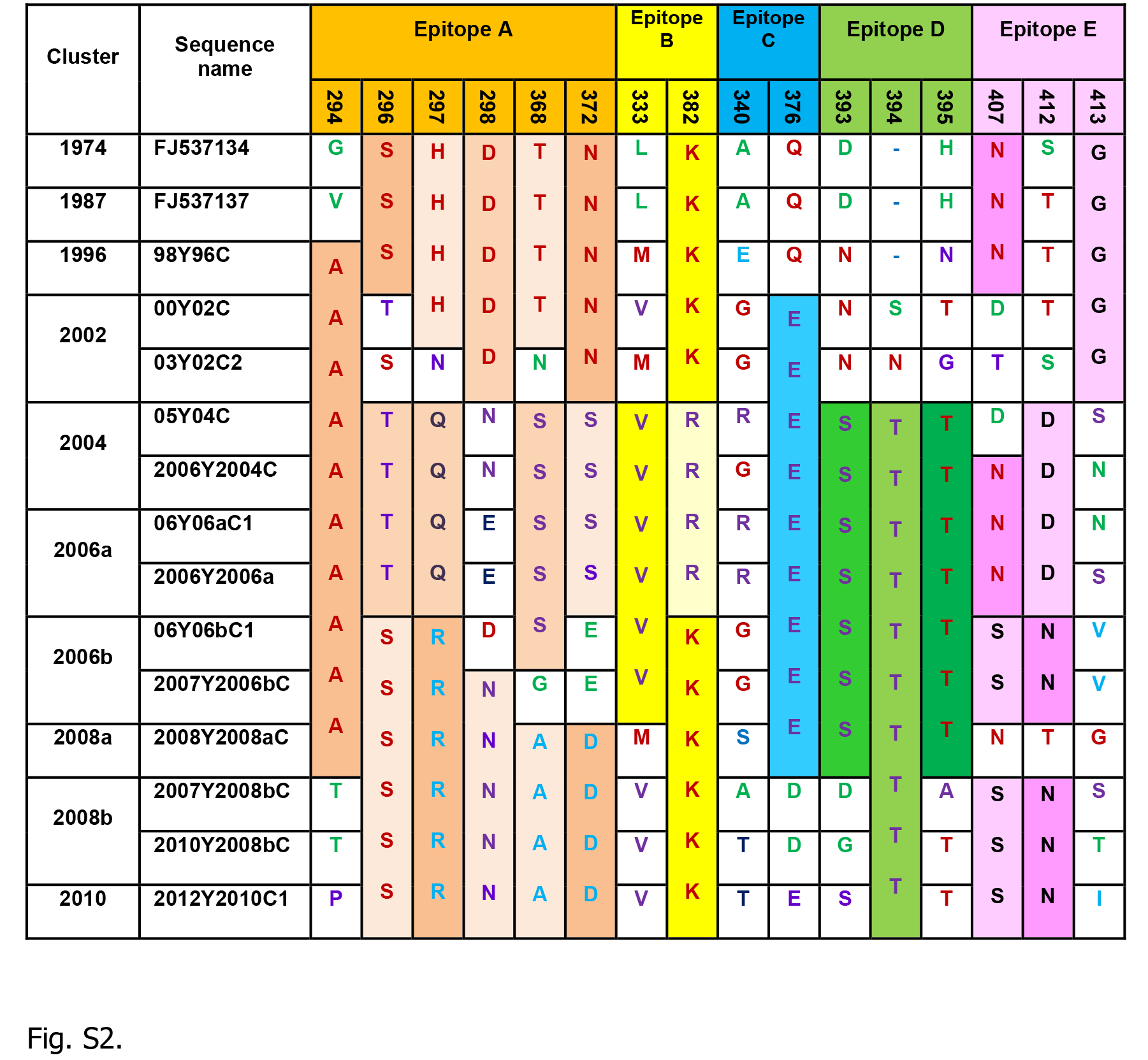

Supplement: S2 Fig — Key sites predicted to modulate receptor binding interactions and the antigenic profiles of the virus were aligned chronologically. (TIF) [file pone.0124945.s002.tif]
