# Supplementary material for: Antigenic Relatedness of Norovirus GII.4 Variants Determined by Human Challenge Sera
Source: PLoS One. 2015 Apr 27;10(4):e0124945. doi: 10.1371/journal.pone.0124945 (PMC4411064; doi:10.1371/journal.pone.0124945)
Supplement: S1 File — (PDF) [file pone.0124945.s003.pdf]

Approval Form

Date:	April 10, 2013
Principal Investigator:	Xiaoli Pang
Study ID:	Pro00037093
Study Title:	Characterization of norovirus strains causing outbreaks in Alberta and seroprevalence study to understand host immune response and fluctuation of outbreak activity in Alberta, Canada
Approval Expiry Date:	April 9, 2014
Funding/Sponsor:	Alberta Innovates Health Solutions

Thank you for submitting the above study to the Health Research Ethics Board - Biomedical Panel. Your application has been reviewed and approved on behalf of the committee.

The Health Research Ethics Board assessed all matters required by section 50(1)(a) of the Health Information Act. It has been determined that the research described in the ethics application is a secondary use of previously collected samples for which subject consent for access to personally identifiable health information would not be reasonable, feasible or practical. Subject consent therefore is not required for access to personally identifiable health information described in the ethics application. In order to comply with the Health Information Act, a copy of the approval form is being sent to the Office of the Information and Privacy Commissioner.

A renewal report must be submitted next year prior to the expiry of this approval if your study still requires ethics approval. If you do not renew on or before the renewal expiry date (April 9, 2014), you will have to re-submit an ethics application.

The membership of the Health Research Ethics Board - Biomedical Panel complies with the membership requirements for research ethics boards as defined in Division 5 of the Food and Drug Regulations and the Tri-Council Policy Statement. The HREB - Biomedical Panel carries out its functions in a manner consistent with Good Clinical Practices.

Approval by the Health Research Ethics Board does not encompass authorization to access the patients, staff or resources of Alberta Health Services or other local health care institutions for the purposes of the research. Enquiries regarding Alberta Health administrative approval, and operational approval for areas impacted by the research, should be directed to the Alberta Health Services Research Administration office, #1800 College Plaza, phone (780) 407-6041.

Sincerely,

J. Stephen Bamforth, MD

Associate Chair, HREB Biomedical

Note: This correspondence includes an electronic signature (validation and approval via an online system).

Animal protocol- three-year renewal, Dr. Jason Jiang, PI, 9-12-13

Our animal research protocol will include three major projects: **1)** characterization of recombinant viral capsid antigens by immunization of mice, guinea pigs and rabbits; **2)** evaluation of the safety of candidate antivirals for norovirus using mouse model; and **3)** evaluation of the safety and efficacy of candidate vaccine against human noroviruses using a gnotobiotic challenge model. Projects 1 and 2 will be performed in Dr. Jiang's laboratory using animal research facilities at the Cincinnati Children's Hospital. Project 3 will be performed by our collaborator, Dr. Lijuan Yuan at the Virginia Tech using their gnotobiotic animal facility. The full protocol and approval of the protocol by the Virginia Tech IACUC is attached in this application.

Project 1. Characterization of recombinant viral capsid antigens by immunization of mice, guinea pigs and rabbits

This animal research protocol will study the immune responses and generation of diagnostic reagents for human caliciviruses (HuCV) by immunization of laboratory animals (mice, rabbits and guinea pigs) with baculovirus-, yeast- and E. coli-expressed recombinant proteins and recombinant adenoviruses carrying human norovirus and rotavirus capsid antigens.

HuCVs are one of the most important viral pathogens causing outbreaks of acute gastroenteritis in children and adults. The human caliciviruses are difficult to study due to the lack of a cell culture and animal model. The reagents and assays developed from this study will be important for large scale epidemiology and clinical diagnosis of human caliciviruses. We also are in the process of developing a vaccine against human noroviruses, the animal immunization study will provide useful information on the efficacy and safety of the vaccine for our future development in clinical trials.

We plan to utilize guinea pigs and rabbits to obtain hyperimmune antisera to HuCV capsid antigens. We will use two 6-week-old Hartley guinea pigs and two specific pathogen-free rabbit per antigen. The hyperimmune antibodies will be used to develop sandwich enzyme immune assays (EIAs) for detection of human calicivirus antigens in stool specimens. For comparison of antigenicity we will immunize a group of mice. The resulting hyperimmune antisera will be compared for antibody titers against HuCVs. We recently developed a dual vaccine containing antigenic epitopes of both noroviruses (NVs) and rotaviruses (RVs), which are potentially useful for protection of children against both NVs and RVs. To characterize this vaccine we also will perform mouse immunization studies using the same procedures described above with an addition of intramuscular immunization.

Antigens used for immunization will be baculovirus-, yeast- or E. coli- expressed HuCV capsid proteins and recombinant adenoviruses carrying human norovirus and rotavirus capsid antigens that will be purified from the cell culture by variable biochemistry methods. For immunization study in mice, variable number of immunization from minimal 2 to maximum 5 may be used to evaluate the immunogenicity of selected viral antigens.

TiterMax (0.5 mL mixed with equal volume of viral antigens per injection for rabbits and guinea pigs and 0.1-0.2 mL mixed with equal volume of viral antigens per injection for mice) will be the major adjuvant used in this study. However, since our previous studies used Freund's complete and incomplete adjuvant was used, we will perform limited number of experiments to include Freund's adjuvant for comparison. The Freund's complete adjuvant will be used for the initial (primary) immunization and incomplete adjuvant used for the second and third immunization.

We will perform three injections at a two-week interval which usually induces a high immune response according to our previous study. For guinea pigs and rabbits, three immunizations will be used. We may give one more injection if a low immune response is obtained, and will not give more injection even if a low response is obtained after the fourth injection.

Project 2. Evaluation of the safety of candidate antivirals for norovirus using mouse model.

We will evaluate the safety of candidate antivirals for norovirus by feeding mice with the candidate compounds followed by observation of clinical sign of from our using mouse model. Briefly, a small number of the most promising compounds (low toxicity and high efficacy in blocking TV infection) will be further tested for tolerance in mice. A fixed-dose procedure as an alternative to the classical 50% lethal dose (LD_{50}) test will be used. Lead compounds will be administered by gavage (up to 0.5 ML) to ICR mice (25±2 g each) (n=10; 5 male, 5 female) at a single dose of one of the 4 fixed-dose levels (5, 50, 500, or 2000 mg/kg of body weight). The objective is to identify a dose that produces clear signs of toxicity but no mortality. Depending on the results of the first test, either no further testing is needed (if no sign of toxicity at 2000 mg/kg, it is considered non-toxic) or a lower dose will be tested. If mortality

occurs, the compound will be retested at a lower dose level (except if the original dose is 5mg/kg). Animals will be observed individually after dosing at least once during the first 30 minutes, periodically during the first 24 hours, with special attention given during the first 4 hours, and daily thereafter, for a total of 14 days, for signs of toxicity, i.e., body weight gain/loss, food and water consumption, body temperature, and gross necropsy (upon euthanasia).

Observations check list: changes in skin and fur, eyes and mucous membranes, and also respiratory, circulatory, autonomic and central nervous systems, and somatomotor activity and behaviour pattern. Attention should be directed to observations of tremors, convulsions, salivation, diarrhea, lethargy, sleep and coma.

All observed signs of toxicity will be scored in relation to the control group. Based on these results, compounds with the best safety profiles will be recommended for future pre-clinical study in collaboration with drug development experts.

Project 3. Evaluation of the safety and efficacy of candidate vaccine against human noroviruses using a gnotobiotic challenge model.

We will evaluate the safety and efficacy of candidate vaccine against human noroviruses using a gnotobiotic challenge model. This project will be performed by our collaborator, Dr. Lijuan Yuan in Virginia Tech. An estimated total of 312 Gn pigs (~60 pigs per year for 5 years) will be needed to complete the project. The following is a brief description of the procedures developed by Dr. Yuan. This protocol was approved by the Virginia Tech IACUC.

1. The rational of the study.
Human noroviruses are the most important cause of pediatric acute gastroenteritis, next to rotaviruses. HuNV is also a major cause of food-borne and water-borne viral gastroenteritis in adult humans, resulting in outbreaks and sporadic cases worldwide. At least 50% of all foodborne outbreaks of gastroenteritis can be attributed to noroviruses. Thus HuNVs have been listed as Category B biological pathogens by the National Institutes of Health/Biodefense Program. Because HuNVs do not grow in cell culture and there is no animal model, except for very recently reported attempts in gnotobiotic pigs, the studies of pathogenesis, host susceptibility, and immunity of HuNVs have been hampered. This study is aimed at developing a gnotobiotic pig model of HuNV infection and disease and to use this model for evaluation of anti-HuNV compounds and study of HuNV protective immunity.

Rotaviruses are also important pathogens causing acute gastroenteritis in children in both developed and developing countries. Each year severe rotavirus gastroenteritis causes 350,000-600,000 deaths in children under 5 years of age. It also accounts for 2 million childhood hospital admissions with an estimated cost of over 1 billion dollars per year. While two new RV vaccines (Rotarix™, GlaxoSmithKline and RotaTeq®, Merck) have been introduced that are safe and highly efficient, there are issues about the vaccines that are not yet fully resolved: 1) Vaccine cost and how widely these vaccines will be distributed into poor countries, where they are most needed. 2) Significantly lower levels of protection of the vaccine in African and some Asian countries. 3) Efficacy against serotype G2 and against a wider range of rotavirus serotypes than those found so far. 4) New circulating strains with new serotypes of RVs may emerge due to the large scale of vaccination of the two vaccines, and 5) severance of virulence of the attenuated vaccines. Thus, studies for the development of non-replicating vaccines are critical for control of RVs.

We recently developed a dual vaccine containing antigenic epitopes of both noro and rotaviruses, which are potentially useful for protection of children against both NVs and RVs. This candidate vaccine now has been selected by the PATH and LigoCyte Pharmaceutics Inc. for further development. We also teamed with the LigoCyte and Virginia Tech to apply an NIH R01 application, in which we propose to evaluate the dual vaccine using Gn pig challenge models for both NVs and RVs. In this animal protocol, we will include human RVs in addition to human NVs in the original protocol. Specifically, we will challenge Gn pigs with both NVs and RVs after vaccination of the animals with the dual vaccines to determine the efficacy of the vaccine in protection against infection and illness caused by both noro and rotaviruses. This study will be performed by our collaborator, Dr. Lijuan Yuan in Virginia Tech. The same animal protocol for the challenge of the human NVs described below will be used.

2. Surgical derivation of pigs.
The sow is restrained in a chute and Ketamine HCl (15 mg/kg) is administered IM via 18g x 1 1/2 in needle. After the sow is sedated, lidocaine (10-15 ml) is given via epidural with 18g x 4 in needle into the Lumbar 6-Sacroiliac 1

space. The sow's hindquarters are elevated and the abdomen is scrubbed with prepodyne and rinsed with bleach (25%). An inhalation bag is placed over the sow's head and CO_2 (70%) is rapidly delivered. After the sow loses consciousness, a ventral midline incision is made. The uterus is exteriorized and the cervix bisected near the body of the uterus. The uterus with fetuses is passed into the transfer tank. The sow is immediately euthanized by electrocution and exsanguination. Piglets are removed from the uterus to a sterile isolator where they are stimulated and the umbilical cords are tied. The gnotobiotic pigs are transferred into sterile rearing isolators.

3. Treatment of pigs.
The Gn pigs will be maintained in germ-free isolation units. Histo-blood group antigens (HBGAs) A+H+ Gn pigs will be identified by EIA using saliva samples collected at 3 days of age. A-H- pigs will be assigned to rotavirus studies (protocol #08-004-CVM). HuNV uses HBGA antigen A and H as receptors to infect pigs. Therefore, A negative and H negative pigs cannot be infected by the virus and have to be excluded from this study. In Aim 1, 40 A+H+ pigs will be randomly assigned to 3 groups (Table 1) receiving a GI (Norwalk virus, GI.1) or a GII (VA387 or #031693, GII.4) virus respectively. In Aims 2 and 3, 90 A+H+ Gn pigs will be randomly assigned into the treatment and control groups (Tables 2 and 3). Pigs will be inoculated orally at 5 days of age (post-inoculation day [PID] 0) with the HuNV strain determined in Aim 1. HBGA oligosaccharide analogues (Aim 2) or lead compounds (Aim 3) will be given orally twice daily to the HuNV-inoculated Gn pigs as prophylactics (Group 1, PID-1 and PID0, before virus inoculation), therapeutics (Group 2, PID1 and PID2), or at both pre- and postinoculation (Group 3, from PID-1 to PID2). All animals in Aims 1-3 will be observed twice daily for signs of illness. Pigs should experience only slight pain or distress at bleedings. Oral inoculation of pigs with HuNV causes a transient diarrhea. HBGA oligosaccharide analogues or lead compounds may prevent or reduce the diarrhea. The safety of the compounds will be evaluated in pigs receiving high dose of compounds for four weeks (Table 3). Pigs will be monitored daily and bled weekly to evaluate overall health conditions (body weight, complete blood cell counts, liver enzymes, and kidney function) and virus-specific antibody and cytokine responses in serum. Pigs with non-responsive anorexia greater than 48 hours or determined moribund (slow or absent righting reflexes) by veterinary examination will be euthanized. If a pig exhibits signs of pain or distress (lethargy, anorexia, hunched posture, respiratory distress), it will be examined by the veterinarian and will be euthanized if not responsive to treatments.

4. Sample collections.
Blood samples will be collected weekly from the jugular vein or anterior vena cava, beginning on the day of inoculation, (using 22 g x 1 in needles and 10 ml syringes). Volumes collected will increase from 0.5 to 2 ml initially (3 to 14 days of age) then to 8 ml in older pigs (>20 days of age). Rectal swab specimens will be collected daily from each pig for 6 days from the date of inoculation. Subsets of pigs will be euthanized at PID3 (8 days of age), 6 (11 days of age) or 28 (33 days of age) to collect jejunum and blood to test for various pathological and immunological parameters (see Table 1).

Animals with non-responsive anorexia of greater than 48 hours duration, or animals determined to be moribund by veterinary examination will be euthanized. Moribund is defined as muscular weakness, loss of coordination or ambulation or a comatose state (non-responsive to touch stimulus and "cold to touch").

Pregnant sows will be purchased from VT Swine Center (Dr. Cindy Wood, Associate Professor and Coordinating Counselor, Department of Animal and Poultry Sciences, 3400 Litton Reaves Hall, Virginia Tech, Blacksburg, VA 24061, 540-231-6936, FAX: 540-231-3010). Hysterectomy and euthanization of the sows and postmortem of the piglets will be performed in the necropsy room (room 4), building 441, CMMID. The room has been renovated and upgraded to meet the requirement. Gnotobiotic pigs will be housed in room C of the Gnotobiotic Pig Facility in building 447A, CMMID. The facility is ready and functional.

Gnotobiotic pigs: Electrocution (ear to ear/transcranial stunning then ear to tail) followed by exsangination (axillary artery) as per the 2007 AVMA Guidelines on Euthanasia. The pigs are not euthanized chemically because mononuclear cells from lymphoid tissues and blood will be harvested from the euthanized pigs for study of immune responses to viral infections. Mononuclear cells isolated from intestinal and systemic lymphoid tissues and blood will be cultured in vitro for 17 hrs to 5 days. It is known that exposure to anesthesia drug, even for short time, can induce immunosuppression, including decreased Th/Tc cell ratio in spleen (Navarro, et al., 1990. Eur Surg Res). Thus, chemicals used for euthanasia will negatively affect the interpretation of the study results. Sows used to derive piglets: Ketamine HCl (100 mg/ml, 15 mg/kg, 24 ml, IM), lidocaine (2%, 22mg/kg, 15 ml, via epidural with 18g x 4 in needle into the Lumbar 6-Sacroiliac 1 space), CO_2 (70% inhalation to effect) followed by electrocution/exsanquination (transcranial stunning, electrocution to effect; and exsanguination via the jugular and axillary artery) as per the the 2007 AVMA Guidelines on Euthanasia. For pigs that are euthanized by electrocution

followed by exsanquination, death is confirmed by observed stop of breathing and heart beat.

The gnotobiotic pig model is a key animal model to investigate infectious agents affecting the gastrointestinal tract (e.g. Norovirus). The use of isolation units permits investigation of pathogenesis of and immune responses to a single strain of viral pathogen, in the absence of contamination with extraneous gut pathogens or gut microflora. Disease symptoms and immune responses exhibited by gnotobiotic pigs closely mimic those observed in humans, making the pigs ideal for studying the pathogenesis of the disease and immune response. Gnotobiotic piglets are derived by a surgical procedure 1 day before the expected delivery date (112 days of pregnancy) into a transfer isolator after which they are transported into rearing isolators where they are kept for the remainder of the experiment. They are housed under environmentally regulated conditions of Hepa filtered air and 90-93 F temperature. Each isolator consists of an entry port, a supply and exhaust filter and two sets of gloves for animal care and manipulation. Isolators are configured to house 1 to 4 pigs, based on the age and size of pigs. Pigs (devoid of maternal antibodies) are fed Parmalat UHT milk or Similac infant formula during the experiment.

Results of literature search

We performed literature search on 9-10-10 using key word “norovirus” “diagnosis” “recombinant proteins” etc. and found 781 references using PubMed from 1981 to current. Most of the references were related to epidemiology of human caliciviruses using ELISA or RT-PCR diagnosis. Some of them also describe development and evaluation of new tests based on recombinant viral antigens which are similar to that we proposed in this protocol. There are also a few references on subunit vaccine candidates for human noroviruses. In conclusion, the areas of norovirus vaccine, immunology and diagnosis remain limited. The use of recombinant capsid antigens to generate antibody and as vaccine candidate remains to be the choice for human caliciviruses.

We did a further literature search on October 11, 2010 in PubMed (http://www.ncbi.nlm.nih.gov/sites/entrez and Scopus (http://www.cincinnatichildrens.org/research/pratt/databases) from 1960 to present date (Oct. 11, 2010) by using terms “Norovirus”, “Vaccine” and “Immunization”. Totally 64 literatures were found in PubMed database and 151 were located in Scopus database. From the published references, we did not find same or related research to those we are performing on using Norovirus recombinant antigens (P particles mainly) as a vaccine candidate, although some studies using Norovirus Virus-Like Particles were found.

We did a third literature search on Nov. 08, 2010 in PubMed (http://www.ncbi.nlm.nih.gov/sites/entrez and Scopus (http://www.cincinnatichildrens.org/research/pratt/databases) from 1966 to present date by using terms (norovirus OR "P-particle") AND (TITLE-ABS-KEY("animal use alternatives" OR "animal testing alternatives" OR anxiolytic OR tranquiliz* OR euthanasia OR euthaniz* OR "tissue culture" OR "cell culture" OR "vitro method" OR "vitro model" OR "vitro technique" OR "alternate model" OR "modified technique") OR TITLE-ABS-KEY("housing modification" OR "modified restraint" OR "computer simulation" OR video OR sofware OR "virtual surgery" OR "virtual reality" OR invertebrate". Totally 165 literatures were found in PubMed database and 132 were located in Scopus database. From the published references, we did not find same or related research to those we are performing on using Norovirus recombinant antigens (P particles mainly) as a vaccine candidate, although some studies using Norovirus Virus-Like Particles were found.

We performed literature search on Aug.30, 2013 in PubMed (http://www.ncbi.nlm.nih.gov/sites/entrez) and Scopus (http://www.cincinnatichildrens.org/research/pratt/databases) using terms (“Norovirus”) AND (TITLE-ABS-KEY (“vaccine” OR “vaccination” OR “animal model” OR “animal use alternatives” OR “immunization”). Totally 279 literatures from 1982 to present date were found in PubMed and 392 literatures from 1993 to present date were located in Scopus. From the published references, we did not find same or related research to those we are performing on using Norovirus recombinant antigens (P particles mainly) as a vaccine candidate.

ARRIVE

The ARRIVE Guidelines Checklist

Animal Research: Reporting In Vivo Experiments

Carol Kilkenny[1], William J Browne[2], Innes C Cuthill[3], Michael Emerson[4] and Douglas G Altman[5]

[1]*The National Centre for the Replacement, Refinement and Reduction of Animals in Research, London, UK,* [2]*School of Veterinary Science, University of Bristol, Bristol, UK,* [3]*School of Biological Sciences, University of Bristol, Bristol, UK,* [4]*National Heart and Lung Institute, Imperial College London, UK,* [5]*Centre for Statistics in Medicine, University of Oxford, Oxford, UK.*

	ITEM	RECOMMENDATION	Section/ Paragraph
Title	1	Provide as accurate and concise a description of the content of the article as possible.	N/A
Abstract	2	Provide an accurate summary of the background, research objectives, including details of the species or strain of animal used, key methods, principal findings and conclusions of the study.	N/A
**INTRODUCTION**			
Background	3	a. Include sufficient scientific background (including relevant references to previous work) to understand the motivation and context for the study, and explain the experimental approach and rationale.<br>b. Explain how and why the animal species and model being used can address the scientific objectives and, where appropriate, the study's relevance to human biology.	N/A
Objectives	4	Clearly describe the primary and any secondary objectives of the study, or specific hypotheses being tested.	N/A
**METHODS**			
Ethical statement	5	Indicate the nature of the ethical review permissions, relevant licences (e.g. Animal [Scientific Procedures] Act 1986), and national or institutional guidelines for the care and use of animals, that cover the research.	√
Study design	6	For each experiment, give brief details of the study design including:<br>a. The number of experimental and control groups.<br>b. Any steps taken to minimise the effects of subjective bias when allocating animals to treatment (e.g. randomisation procedure) and when assessing results (e.g. if done, describe who was blinded and when).<br>c. The experimental unit (e.g. a single animal, group or cage of animals).<br>A time-line diagram or flow chart can be useful to illustrate how complex study designs were carried out.	N/A
Experimental procedures	7	For each experiment and each experimental group, including controls, provide precise details of all procedures carried out. For example:<br>a. How (e.g. drug formulation and dose, site and route of administration, anaesthesia and analgesia used [including monitoring], surgical procedure, method of euthanasia). Provide details of any specialist equipment used, including supplier(s).<br>b. When (e.g. time of day).<br>c. Where (e.g. home cage, laboratory, water maze).<br>d. Why (e.g. rationale for choice of specific anaesthetic, route of administration, drug dose used).	√
Experimental animals	8	a. Provide details of the animals used, including species, strain, sex, developmental stage (e.g. mean or median age plus age range) and weight (e.g. mean or median weight plus weight range).<br>b. Provide further relevant information such as the source of animals, international strain nomenclature, genetic modification status (e.g. knock-out or transgenic), genotype, health/immune status, drug or test naïve, previous procedures, etc.	√

The ARRIVE guidelines. Originally published in *PLoS Biology*, June 2010[1]

Housing and husbandry	9	Provide details of:<br>a. Housing (type of facility e.g. specific pathogen free [SPF]; type of cage or housing; bedding material; number of cage companions; tank shape and material etc. for fish).<br>b. Husbandry conditions (e.g. breeding programme, light/dark cycle, temperature, quality of water etc for fish, type of food, access to food and water, environmental enrichment).<br>c. Welfare-related assessments and interventions that were carried out prior to, during, or after the experiment.	✓
Sample size	10	a. Specify the total number of animals used in each experiment, and the number of animals in each experimental group.<br>b. Explain how the number of animals was arrived at. Provide details of any sample size calculation used.<br>c. Indicate the number of independent replications of each experiment, if relevant.	✓
Allocating animals to experimental groups	11	a. Give full details of how animals were allocated to experimental groups, including randomisation or matching if done.<br>b. Describe the order in which the animals in the different experimental groups were treated and assessed.	N/A
Experimental outcomes	12	Clearly define the primary and secondary experimental outcomes assessed (e.g. cell death, molecular markers, behavioural changes).	✓
Statistical methods	13	a. Provide details of the statistical methods used for each analysis.<br>b. Specify the unit of analysis for each dataset (e.g. single animal, group of animals, single neuron).<br>c. Describe any methods used to assess whether the data met the assumptions of the statistical approach.	N/A
**RESULTS**			
Baseline data	14	For each experimental group, report relevant characteristics and health status of animals (e.g. weight, microbiological status, and drug or test naïve) prior to treatment or testing. (This information can often be tabulated).	N/A
Numbers analysed	15	a. Report the number of animals in each group included in each analysis. Report absolute numbers (e.g. 10/20, not 50%[2]).<br>b. If any animals or data were not included in the analysis, explain why.	✓
Outcomes and estimation	16	Report the results for each analysis carried out, with a measure of precision (e.g. standard error or confidence interval).	✓
Adverse events	17	a. Give details of all important adverse events in each experimental group.<br>b. Describe any modifications to the experimental protocols made to reduce adverse events.	N/A
**DISCUSSION**			
Interpretation/ scientific implications	18	a. Interpret the results, taking into account the study objectives and hypotheses, current theory and other relevant studies in the literature.<br>b. Comment on the study limitations including any potential sources of bias, any limitations of the animal model, and the imprecision associated with the results[2].<br>c. Describe any implications of your experimental methods or findings for the replacement, refinement or reduction (the 3Rs) of the use of animals in research.	N/A
Generalisability/ translation	19	Comment on whether, and how, the findings of this study are likely to translate to other species or systems, including any relevance to human biology.	N/A
Funding	20	List all funding sources (including grant number) and the role of the funder(s) in the study.	✓

NC 3Rs

References:
1. Kilkenny C, Browne WJ, Cuthill IC, Emerson M, Altman DG (2010) Improving Bioscience Research Reporting: The ARRIVE Guidelines for Reporting Animal Research. *PLoS Biol* 8(6): e1000412. doi:10.1371/journal.pbio.1000412
2. Schulz KF, Altman DG, Moher D, the CONSORT Group (2010) CONSORT 2010 Statement: updated guidelines for reporting parallel group randomised trials. *BMJ* 340:c332.
